# In Situ Synthesis of Hybrid Inorganic–Polymer Nanocomposites

**DOI:** 10.3390/polym10101129

**Published:** 2018-10-11

**Authors:** Mohammed M. Adnan, Antoine R. M. Dalod, Mustafa H. Balci, Julia Glaum, Mari-Ann Einarsrud

**Affiliations:** 1 Department of Materials Science and Engineering, Norwegian University of Science and Technology, 7491 Trondheim, Norway; mohammed.m.adnan@ntnu.no (M.M.A.); julia.glaum@ntnu.no (J.G.); 2 poLight ASA, Kongeveien 77, 3188 Horten, Norway; antoine.dalod@polight.com; 3 Department of Physics, Norwegian University of Science and Technology, 7491 Trondheim, Norway; mustafa.h.balci@ntnu.no

**Keywords:** nano-hybrids, nanocomposites, sol–gel, in situ synthesis, metal oxides

## Abstract

Hybrid inorganic–polymer nanocomposites can be employed in diverse applications due to the potential combination of desired properties from both the organic and inorganic components. The use of novel bottom–up in situ synthesis methods for the fabrication of these nanocomposites is advantageous compared to top–down ex situ mixing methods, as it offers increased control over the structure and properties of the material. In this review, the focus will be on the application of the sol–gel process for the synthesis of inorganic oxide nanoparticles in epoxy and polysiloxane matrices. The effect of the synthesis conditions and the reactants used on the inorganic structures formed, the interactions between the polymer chains and the inorganic nanoparticles, and the resulting properties of the nanocomposites are appraised from several studies over the last two decades. Lastly, alternative in situ techniques and the applications of various polymer–inorganic oxide nanocomposites are briefly discussed.

## 1. Introduction

Hybrid inorganic–polymer materials have been studied extensively over the last 30 years due to the unique combination of properties that can arise, especially when the inorganic domains possess a dimension in the nanoscale (below 100 nm), forming a nanocomposite [1,2,3,4,5,6]. The potential combination of the advantages of inorganic materials (e.g., high hardness, high thermal stability, high refractive index, chemical stability, etc.) with those of organic polymers (e.g., processability, flexibility, low weight, etc.) can enable a wide range of applications for these nanocomposites. These range from common plastics reinforcement to abrasion resistant coatings [2,7], flame-retardant materials [8,9], catalysis [10,11], memory devices [12], integral capacitors [13], optical devices [14,15], electrical insulation in microelectronics and nanodielectrics [16,17,18], fuel cells [19], etc. The defining feature of nanocomposites is the larger interfacial area of the nanoscale inorganic fillers, in comparison to traditional composites. This larger interfacial area results in a considerable volume of interfacial polymer, for a lower filler content, with properties that are unique from the bulk polymer [2,20,21]. The emergence of these new properties can be attributed in part to the interactions between the organic and inorganic components at the interface. Hybrid materials can be divided into two classes, based on the nature and strength of these interactions: In Class I hybrids, there are only weak bonds (e.g., van der Waals, or hydrogen bonds) between the organic and inorganic components, while in Class II hybrids, strong chemical bonds are prevalent at the interfaces [4,7,22]. These interactions are correlated to the size, shape, size distribution, and dispersion state of the nanoparticle fillers. However, nanoscale materials have a tendency to agglomerate in order to minimize the high surface energy [23,24]. The agglomeration of nanoparticles will reduce the interfacial area and the interactions with the polymers in nanocomposites, thereby negating the potential benefits of using nanoscale fillers. In some cases, agglomeration may even result in the deterioration of material properties and act as defects in the system.

One of the primary challenges in the synthesis of nanocomposites is to ensure a homogeneous dispersion of the inorganic fillers in the polymer matrix. In standard top–down ex situ methods, nanoparticles are pre-synthesized and then mixed into the polymer (blending or intercalation) or a monomer (followed by in situ polymerization) [15,25]. Any agglomerates formed during the synthesis of these nanoparticles (e.g., during flame pyrolysis or precipitation) are difficult to break up during this mixing due to the viscosity of the polymer [26,27]. The resulting inhomogeneity in the nanocomposites can deteriorate the properties of the material. Surface functionalization of the nanoparticles may help to prevent agglomeration of the nanoparticles, or reduce any phase separation due to the incompatibility between the hydrophilic inorganic fillers and the hydrophobic polymer matrix [23,26]. The surface modification can be done using either physical interactions (e.g., surfactants or adsorbed macromolecules) [2,28,29] or chemical interactions (e.g., silane coupling agents or grafted ligands), resulting in Class I and Class II hybrid materials, respectively. However, even with the use of surface-functionalized nanoparticles, it is challenging to achieve a homogeneous dispersion of non-agglomerated nanoparticles in the polymer matrix through conventional ex situ mixing techniques.

An alternative approach to achieve a homogeneous dispersion is the in situ synthesis of inorganic nanoparticles in the polymer matrix, using techniques such as sol–gel chemistry, reverse microemulsion, or hydrothermal/solvothermal synthesis [2,3,7,15,22,23,24,26,30,31]. These methods typically involve the mixing of precursors with a non-reactive solvent and the monomer/polymer, where the reaction of the precursors initiates the synthesis of particles either before or during polymerization [23,27]. This bottom–up approach to the preparation of nanocomposites can enable increased control over the structure and properties of the nanocomposite by incorporating particle generation, surface modification, and integration into the polymer matrix in one process. Since the nanoparticles are nucleated and grown inside the polymer matrix, the passivating effect of the polymer chain functional groups on the nanoparticles can control particle size and reduce agglomeration [15]. One limitation of such an approach, however, is that the unreacted precursors or byproducts of the in situ reactions may alter the properties of the nanocomposite.

Due to the numerous works published on the in situ preparation of many different nanocomposites, this article aims to familiarize the reader with the sol–gel method for in situ synthesis of inorganic oxides in polymers. SiO_2_ (silica) and TiO_2_ (titania) are two of the most common inorganic oxide nanofillers used in hybrid materials, and are featured more heavily in this work. A brief synopsis of several other in situ techniques for fabrication of nanocomposites is also provided at the end. In addition to the development of the techniques and strategies used, a discussion of the properties and relevant applications for such nanocomposites is included. For polymer systems, epoxy resins and poly(dimethylsiloxane) are focused on in this review, due to their versatility in multiple areas of application, such as in laminates, structural composites, electrical insulation, and coatings for epoxy [32], and in coatings, optical devices (e.g., LED encapsulation and optical waveguides) and bioactive materials for polydimethylsiloxane (PDMS) [14,33].

## 2. The Chemistry of In Situ Reactions

### 2.1. Sol–Gel Process and the Formation of the Inorganic Network

**Figure 1:**
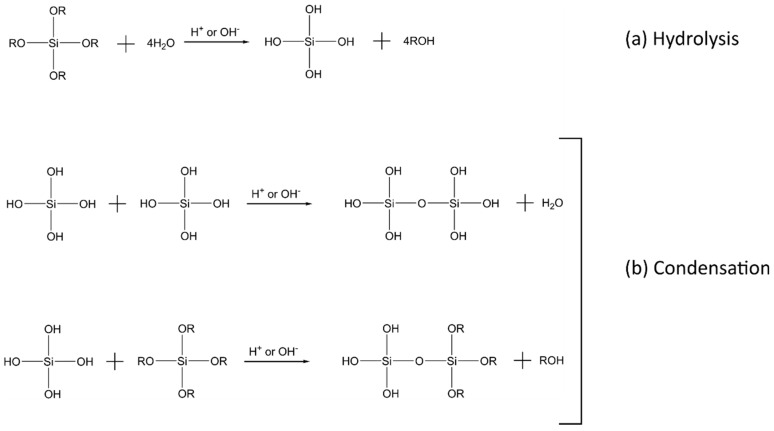
The (**a**) hydrolysis; and (**b**) condensation reactions of a silicon alkoxide precursor (Si(OR)_4_) in a sol–gel process.

Sol–gel reactions have been used extensively in the preparation of inorganic materials (e.g., glasses and ceramics), and is one of the most common routes for preparing amorphous hybrid networks in situ at low temperatures [2,3,15,27,30,34]. The sol–gel process is well described and consists of two steps: First, the hydrolysis of a molecular precursor (typically a metal alkoxide), followed by a polycondensation reaction to form the inorganic network (Figure 1). Both reactions can also occur simultaneously once hydrolysis has been initiated. The reactions can be summarized by the following equation, where M represents a metal and R an alkyl group, and X represents H during hydrolysis and M during condensation [35]:

M(OR)_n_ + *m*XOH → [M(OR)*_n-m_* (OX)*_m_*] + *m*ROH (1)

This method is of particular interest in the in situ formation of an inorganic network (e.g., SiO_2_, TiO_2_, ZnO, Al_2_O_3_, ZrO_2_, etc.) in a polymer matrix, via the swelling of a polymeric host by a solution containing the precursors (e.g., tetraethylorthosilicate (TEOS) and titanium (IV) isopropoxide (TIP)), followed by promotion of the sol–gel reactions [27]. The solvent used is an important parameter for controlling the polymer solubility and preventing liquid–liquid phase separation. Commonly used solvents include alcohols, tetrahydrofuran (THF), and *N*,*N*-dimethylformamide (DMF) [2,22,36].

**Figure 2:**
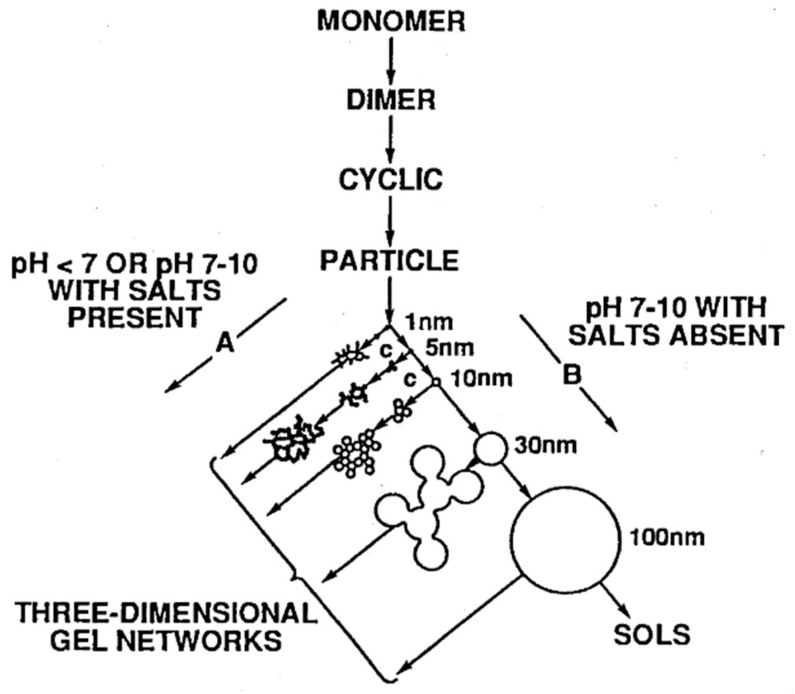
The polymerization behavior of aqueous silica from silicon alkoxide precursors in basic and acidic solutions with and without flocculating salts. Reproduced with permission from Reference [40].

Several parameters can be adjusted to control the size and morphology of the materials formed by sol–gel processes. The reactivity of the metal alkoxide (which is affected by the type of metal and the steric hindrance of the alkoxy groups) will affect the rate of the hydrolysis reaction, which in turn affects the structure of the metal–oxo networks that form. The hydrolysis is faster when the metal cation has high electrophilicity and high degree of unsaturation (*N* − *Z*, where *N* is the coordination number and *Z* is the oxidation state of the metal) [4]. Transition metal alkoxides (e.g., M(OR)_4_, where M = Ti, Sn, Zr, Ce, etc.) are therefore typically very reactive (since *N* − *Z* > 0 typically) [4,22,27], and hydrolyze very easily in the presence of even a small amount of moisture. As a result, inhibitors may be required to prevent precipitates from forming before the condensation reactions can occur, such as chelating agents that stabilize the alkoxide and reduce reactivity (e.g., by increasing steric hindrance). Silicon alkoxides, on the other hand, are less reactive due to their low electrophilicity [4] and require a catalyst to increase the hydrolysis reaction rate [2,22,27]. Acid catalysis promotes the hydrolysis reaction, resulting in the formation of open structures with low fractal dimension [27,30]. Base catalysis, meanwhile, promotes the condensation reaction, leading to colloidal particulate structures [27,30]. Figure 2 shows how pH affects the polymerization behavior of silicon alkoxides.

Larger alkoxy groups have greater steric hindrance, resulting in a stabilizing effect that reduces the reactivity of the metal alkoxide. The kinetics of the hydrolysis and condensation reactions are also affected by the temperature, ratio of water to metal alkoxide, type of solvent, etc. [2,30,34]. Control of the reactivity of metal alkoxides is particularly important when two or more of them must be combined in one polymer system (e.g., in the preparation of nanocomposites with several inorganic oxides as filler), in order to prevent phase separation or precipitation.

In addition, the sol–gel process can also be non-hydrolytic, that is, the process is solvent-free and does not require a water catalyst. The non-hydrolytic sol–gel (NHSG) route usually involves a reaction between a metal precursor and an oxygen donor (e.g., alkoxide, ether, alcohol, carboxylates, etc.) under non-aqueous conditions to form the inorganic oxide [37,38]. The reaction proceeds via a ligand exchange mechanism that is catalyzed by Lewis acids [39]. Due to the difference in reaction mechanism, the reactivity differences for different metals seen earlier in the hydrolytic process may not be the same in this case. The NHSG route offers an alternative in cases where the conventional hydrolytic sol–gel synthesis routes may not be optimal. It is easier to control, as the reactions are slower than for hydrolytic sol–gel [38,39]. Some common reaction pathways in NHSG methods include alkyl halide elimination (reaction between metal chloride and metal alkoxide), ester elimination (reaction between metal alkoxides and acetates), and ether elimination (condensation reaction between metal alkoxides) [38].

### 2.2. Formation of the Organic Network and Crosslinks between the Organic and Inorganic Components in Hybrids

The in situ formation of nanoparticles or an inorganic network via sol–gel methods may occur either in the presence of a preformed polymer (that is already polymerized), or by simultaneous formation of both the organic and inorganic networks, forming an interpenetrating polymer network (IPN) [2]. While the inorganic network in hybrids is formed via hydrolysis and condensation reactions, the organic network is formed via polymerization reactions between the monomers, forming macromolecules with repeating units [41,42]. The polymers may be classified into two basic types: Addition and condensation polymers.

**Figure 3:**
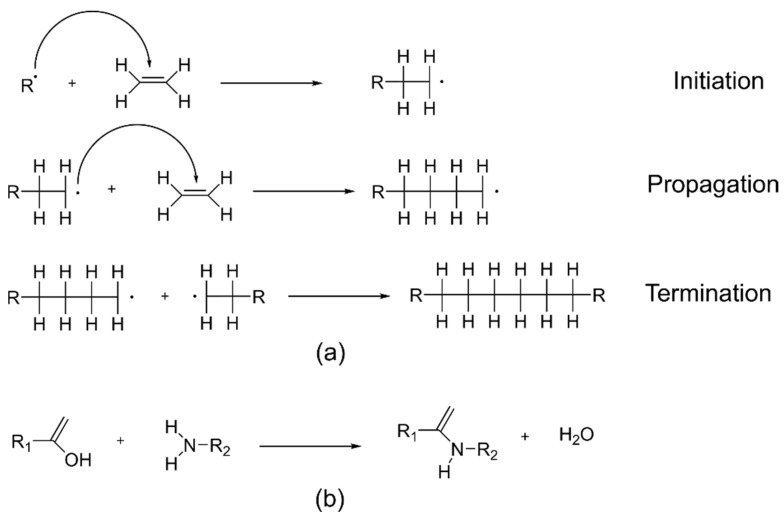
Reactions for the formation of (**a**) addition polymers (showing radical polymerization with one of the possible termination steps); and (**b**) condensation polymers. R· represents a free radical, while R_1_ and R_2_ represent two different organic groups.

Figure 3 shows example reactions in the formation of these two types of polymers. Addition polymers are formed by the linking of monomers without the formation of any byproducts. Addition reactions may be initiated by free radicals, and propagated by the chain radicals (as shown in Figure 3a). Reactions between radicals or radical transfer reactions can terminate radical polymerization. Alternatively, Ziegler–Natta catalysts may also be used in the synthesis of addition polymers [42]. Polyethylene and polypropylene are common examples of addition polymers. Unlike addition polymers, condensation polymers are typically synthesized using difunctional monomers, or different monomers with end groups that can react with each other to form the chain. As a result, a small molecular byproduct (e.g., water, methanol, etc.) may also form (as shown in Figure 3b). Branches or crosslinks can form if a trifunctional monomer is present in addition. Polyamides and polyesters are two classes of polymers that form via condensation reactions.

The properties of nanocomposites prepared by the sol–gel process are affected by the size of the particles formed, as well as the interactions between the inorganic and organic components. Strong chemical bonds between the continuous and dispersed phases resulting in the formation of Class II hybrid materials are preferred, since the presence of these bonds will facilitate dispersion and reduce phase separation. However, for these bonds to form, there must be suitable functional groups available on the polymer chains. In some cases, there may be competition for the bonds to form, as these functional groups generally also react with functional groups on other monomers in order to increase the chain length. In other cases, coupling agents may be used to form bridges between the inorganic domains with either the polymer chains or monomer units when there are no suitable functional groups available for bond formation with the inorganic components.

Silane coupling agents (SCAs) are one such example and are often used for modifying the surfaces of filler particles in nanocomposites to increase compatibility between the organic and inorganic components [28,29,43]. These are organosilicon compounds with two different functional groups, typically with the formula X(CH_2_)_n_SiR_3_, where X is a functional organic group and R is a hydrolysable group [29,43,44]. The organic group reacts with the polymer matrix and the hydrolysable group reacts with the surface of the inorganic nanoparticles. Commonly used SCAs include 3-aminopropyltriethoxysilane (APTES), 3-glycidyloxypropyltrimethoxysilane (GPTMS), 3-isocyanatopropyltriethoxysilane (IPTES), *n*-decyltriethoxysilane (DTES), and methacryloxypropyltrimethoxysilane (MPTMS) [28,29,45,46,47,48,49,50,51]. The SCAs may be introduced to the nanocomposites via several paths, including copolymerization with the monomers and reaction with the preformed polymer or the silicon precursor (or a mixture of the two). Modification of the preformed polymer by the SCA before the sol–gel process is the frequently used approach [2,48,49,50,51,52], allowing polycondensation reactions between the trialkoxysilyl groups on the SCA bonded to the polymer and the metal alkoxide precursor, forming a covalent bond between the two phases.

In addition to SCAs, other coupling agents include carboxylic acids (e.g., oleic acid, tetrafluorobenzoic acid, etc.), polymer/copolymer chains (e.g., poly(ethylene glycol), polymethyl methacrylate, poly(glycidyl methacrylate), etc.), and organophosphorus molecules (e.g., phosphonic acids, aminophenyl phosphate, etc.) [2,28,29,53].

## 3. Nanocomposite Fabrication via Sol–Gel Processes

A comprehensive overview over all the inorganic–polymer nanocomposites prepared by an in situ sol–gel synthesis is beyond the scope of this review. Therefore, selected examples on the development of epoxy and polydimethylsiloxane (PDMS) nanocomposites prepared using sol–gel processes will be presented. The inorganic components of the nanocomposites from these examples are primarily transition metal oxides (e.g., TiO_2_, ZrO_2_, etc.) or silica (SiO_2_). Table 1 shows a general overview of the various syntheses of nanoparticles in situ in the two different polymer systems. It should be noted that in some of the works referenced, the authors do not specify the inorganic component in the hybrids as nanoparticles, but instead as nanodomains. This is most likely because the inorganic networks formed are so small and polymer-like that they may not qualify as particles with a defined shape (e.g., spheres). This is more prevalent in the works on PDMS nanocomposites, where the hybrids are called, for example, M–O–PDMS (where M is the transition metal) instead of PDMS–M_x_O_y_ nanocomposites. In Table 1, the inorganic components specified are based on the assumption that these inorganic networks will form nanoparticles if they grow to an appreciable size. The chemistry behind the synthesis routes, the effect of various parameters on the inorganic structures formed, as well as the resulting properties of the nanocomposites are reviewed afterwards.

### 3.1. Epoxy Nanocomposites

Epoxy is a thermosetting polymer and an excellent choice for high performance composite materials when reinforced with SiO_2_ due to the resulting strength, toughness, good chemical and heat resistance, and high thermal stability [48,49,51]. Typically, epoxy composites are cured via a condensation reaction with an amine- or anhydride-based curing agent, forming a copolymer. Epoxy nanocomposites containing titania (TiO_2_) are also of interest due to the photocatalytic properties imparted to the polymer by the TiO_2_, as well as increases in the refractive index [15,60,61]. Due to the challenges with achieving a homogeneous dispersion of nanoparticles when employing a traditional ex situ blending route, there has been an increased focus on the use of in situ sol–gel techniques instead for nanocomposite synthesis. Diglycidyl ether of bisphenol A (DGEBA) is commonly used as the monomer, and poly(oxypropylene diamine), also known as Jeffamine, is often used as the curing agent in these nanocomposites.

**Figure 4:**
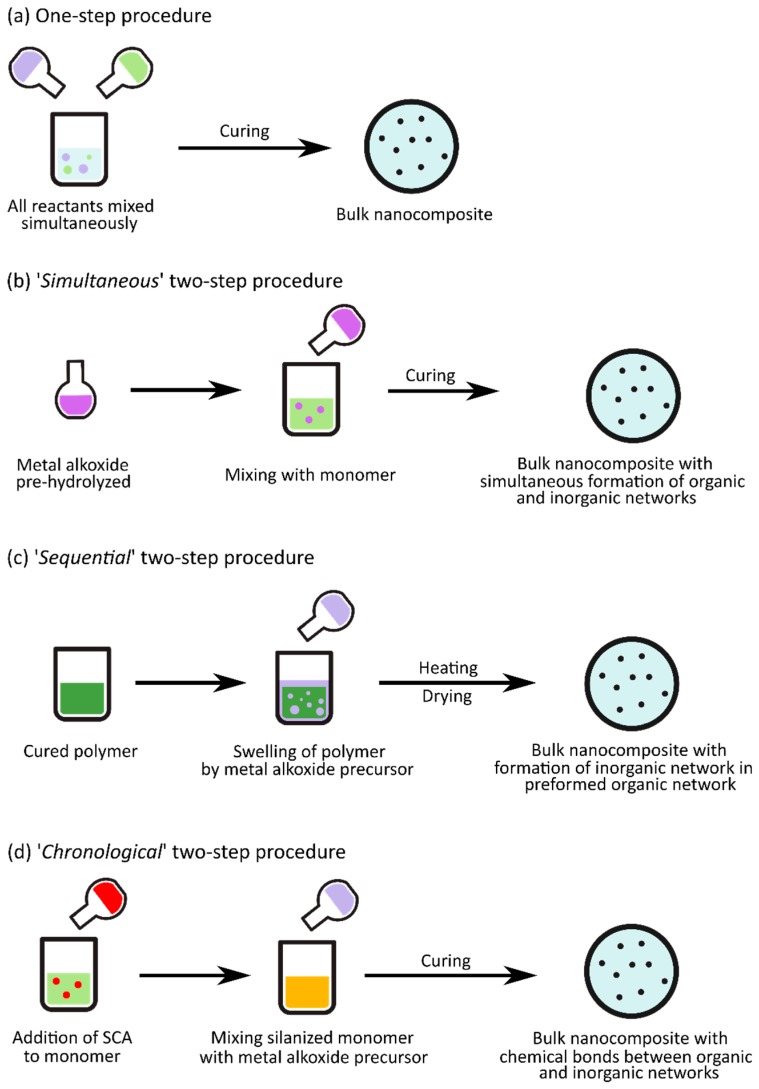
Schematic displaying the general principles of the (**a**) one-step procedure; and the (**b**) ‘s*imultaneous*’; (**c**) ‘s*equential*’; and (**d**) ‘c*hronological*’ two-step procedures used in the in situ synthesis of epoxy nanocomposites. The colors of the various solutions are described as follow: The pure epoxy resins are indicated either by light green (monomer solution), dark green (polymer solution), or orange (monomers modified by silane coupling agents (SCA)), the inorganic oxide precursors are indicated by light purple, or dark purple (pre-hydrolyzed), and the SCAs are indicated by red.

For synthesizing nanoparticles in situ in epoxy, most researchers have attempted either a one-step or a two-step procedure, as shown in Figure 4. In the one-step procedure, the precursors and reaction components (epoxy resin, coupling agent, inorganic oxide precursor, curing agent, solvent, catalysts, etc.) are all mixed simultaneously and reacted, before being cast into bulk films. There are several variations of the two-step procedure. In a ‘*simultaneous*’ two-step procedure, the inorganic oxide precursor (TEOS, TIP, etc.) is pre-hydrolyzed in the first step using a catalyst (e.g., *p*-toluenesulfonic acid monohydrate (TSA) or dibutyltin dilaurate (DBTDL)). The second step involves the polymerization of the organic components and the formation of the oxide network simultaneously when the pre-hydrolyzed precursor is mixed with the monomer and curing agent. In a ‘*sequential*’ two-step procedure, the epoxy resin is cured in the first step, before being swollen by the alkoxide, water, catalysts, etc. in the second step. The inorganic oxide network in this case forms in a preformed organic network, as the epoxy is already cured. Finally, there is also the ‘*chronological*’ two-step procedure where an SCA is first added to the epoxy to form modified (silanized) monomer chains. In the next step, the inorganic precursors (alkoxide, water, catalyst, etc.) are added sequentially to form the oxide network before the nanocomposite is cured. Since the coupling agents provide a chemical bond between the organic and inorganic networks, this procedure results in the formation of Class II hybrids. One of the advantages with a two-step procedure is that it offers more control over specific reactions, depending on which variation of the procedure is used, since not all of the reactions are occurring simultaneously, as in the one-step procedure.

#### 3.1.1. Effect of Synthesis Procedure and pH on the Structure and Morphology of Nanocomposites

Matějka et al. prepared epoxy–SiO_2_ nanocomposites using a one-step procedure [55,56,57], a simultaneous two-step procedure [55,56,57], and a sequential two-step procedure [56,57]. Differences in the structure of the inorganic domains arose based on whether the reaction was carried out in a one-step or two-step procedure. In the one-step procedure, large SiO_2_ aggregates (100–300 nm) were observed through scanning electron microscopy (SEM) [57], which was attributed to the reaction being catalyzed by the amine curing agent (a base) due to its molar excess over the acidic catalyst (TSA). Base catalysis promotes the condensation reaction and the formation of colloidal (spherical) particles. Small angle X-ray scattering (SAXS) experiments revealed compact silica structures with high fractal dimension (*D*_m_ = 2.7) [55,57]. For hybrids prepared using the two-step simultaneous process, smaller SiO_2_ structures were observed (50–100 nm) with a lower fractal dimension (*D*_m_ = 1.7), indicating a more open SiO_2_ cluster due to the TEOS being pre-hydrolyzed by an acid [55,56,57]. The choice of catalyst can also affect the morphology—DBTDL was seen to be less effective at hydrolyzing TEOS than TSA, resulting in more compact SiO_2_ clusters (*D*_m_ = 2.5–2.7) [56,57]. In the two-step sequential process, the distribution of the inorganic phase was not uniform, with a higher SiO_2_ concentration on the surface. This was due to the inhomogeneous swelling of the epoxy resin by the TEOS. However, the SiO_2_ domains were small (10 nm) and formed an open structure (*D*_m_ = 1.9–2.2) due to the acid catalysis of the TEOS hydrolysis [57]. Dynamic mechanical analysis (DMA) showed a larger shear storage modulus for the in situ epoxy-SiO_2_ nanocomposites compared to pure epoxy [57]. However, this reinforcement was dependent on the procedure used for preparation. Acid pre-hydrolysis of TEOS resulted in higher modulus in the nanocomposites, compared to those prepared without pre-hydrolysis (e.g., in the one-step procedure or when the TEOS was pre-hydrolyzed by pH neutral DBTDL catalyst). The sequential two-step procedure with pre-hydrolyzed TEOS possessed the largest storage modulus. In addition, the loss factor (tan δ) also decreased and broadened with the inclusion of SiO_2_ in epoxy, with the sequential two-step prepared hybrid showing the largest decrease. The observed reinforcement effects are attributed to increasing interphase interactions in the hybrid systems, resulting in a larger immobilized layer of polymer chains around the nanoparticles [57]. The nanocomposites were determined to have a bicontinuous morphology (the SiO_2_ forms a continuous phase in the organic matrix) rather than a particulate composite (with dispersed SiO_2_ particles), based on agreement of the data with the two different models [57].

Bauer et al. [54] similarly prepared epoxy–SiO_2_ nanocomposites using both a one-step procedure and a two-step sequential procedure, but without any additional catalysts. SAXS data (corroborated by TEM images) showed that the nanocomposites prepared using a one-step procedure had extensive phase mixing (slope of −2 in the Porod region), while those prepared using the two-step sequential procedure (with the pre-cured epoxy) were strongly phase-separated (slope of −4 in the Porod region) [54]. The latter result is contrary to that presented by Matějka et al. [56], where the sequential procedure also led to phase mixing (−2 slope in the Porod region). This difference was attributed by Matějka et al. [56] to differences in the temperature of the synthesis (60 °C instead of 90 °C), with a higher temperature promoting increased grafting between the organic and inorganic networks. Thermogravimetric analysis (TGA) also showed increased thermal stability for the nanocomposites, with the initial mass loss occurring at 20–50 °C higher temperatures than for pure epoxy resin [54]. A decrease in the slope of the thermogravimetric curves (resembling a small plateau) was observed between 400 and 600 °C for pure epoxy resin, corresponding to char formation. This plateau was shifted to higher temperatures for the nanocomposites, with the inorganic network possibly acting as a barrier to the decomposition of the organics. The skeleton-like morphology of the SiO_2_ remaining after the organic burn-off indicated the formation of an interpenetrating polymer network (IPN), similar to the bicontinuous morphology suggested by Matějka et al. [57].

#### 3.1.2. The Effect of Silane Coupling Agents

**Figure 5:**
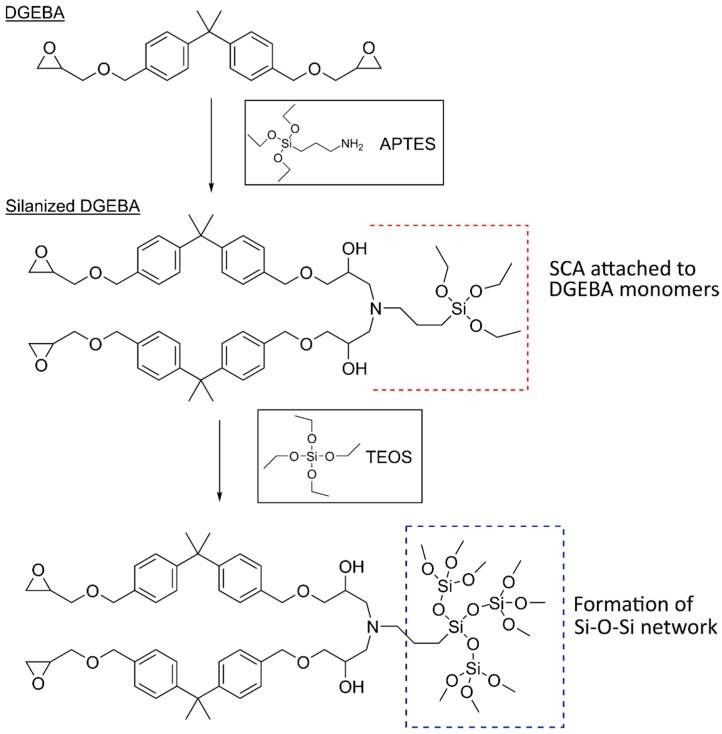
Schematic of the in situ sol–gel reactions possibly occurring in the ‘chronological’ two-step procedure for the preparation of epoxy–SiO_2_ nanocomposites. Diglycidyl ether of bisphenol A (DGEBA) is used as the epoxy monomer, 3-aminopropyltriethoxysilane (APTES) as the coupling agent, and tetraethylorthosilicate (TEOS) as the silica (SiO_2_) precursor. The curing step is not shown in this schematic.

Several works have also employed the ‘chronological’ two-step procedure in the preparation of epoxy nanocomposites, using SCAs to improve the dispersion of the nanoparticles formed in situ. Figure 5 shows a schematic for a possible outline of the reactions occurring during this procedure between the DGEBA monomer, the coupling agent, and the precursor.

**Figure 6:**
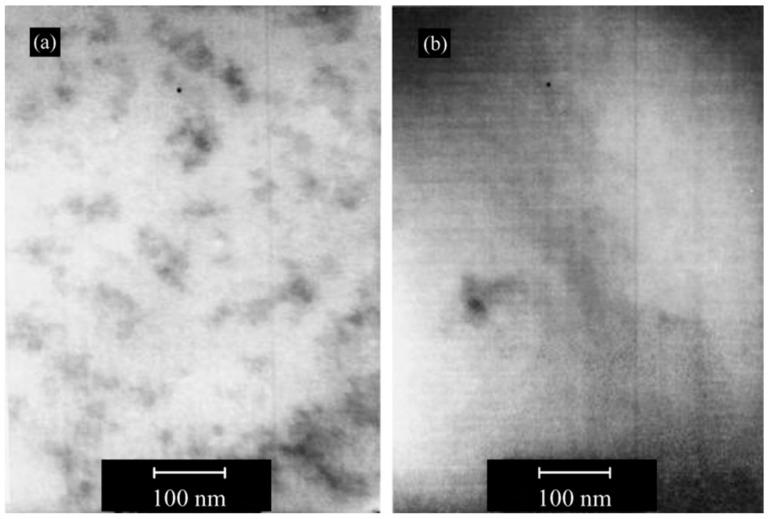
TEM micrographs of epoxy–SiO_2_ hybrids with 10 wt % SiO_2_: (**a**) Hybrid films prepared without SCAs; and (**b**) hybrid films prepared with APTES. Reproduced with permission from Nazir et al. *Progress in Organic Coatings*; published by Elsevier B.V., 2010.

Nazir et al. [52] prepared epoxy–SiO_2_ nanocomposites by first modifying the DGEBA monomer with the SCA APTES, followed by sol–gel reaction with TEOS and water and subsequent curing using Jeffamine. Nanocomposites were also prepared without the SCA using the same synthesis procedure, minus the addition of the APTES. TEM images (Figure 6) showed clear differences between the samples (with the same SiO_2_ content) prepared with and without the SCA. The nanocomposites without the SCA showed distinct SiO_2_ particles, indicating a two-phase morphology, whereas the nanocomposites with the SCA showed less distinct organic and inorganic phases, representing a bicontinuous phase morphology similar to that proposed by Matějka et al. [57]. TGA also showed that the thermal stability, as well as the average energy of activation (*E*_a_) for the degradation, slightly increased for the nanocomposites when APTES was used [52]. However, there was no indication of char formation. Dynamic mechanical thermal analysis (DMTA) showed a higher storage modulus for the nanocomposites in the glassy region. The storage modulus increased with increasing SiO_2_ content up to 10 wt % for samples prepared without APTES and up to 15 wt % for samples prepared with APTES (which also showed the highest storage modulus). Further increase in the SiO_2_ content led to a decrease in the storage modulus. The glass transition temperature (*T*_g_), given by the position of the loss factor peak, also increased with increasing SiO_2_ content (by approximately 4 °C up to 10 wt %) [52]. *T*_g_ was observed to be higher for samples prepared with APTES than for those without.

**Figure 7:**
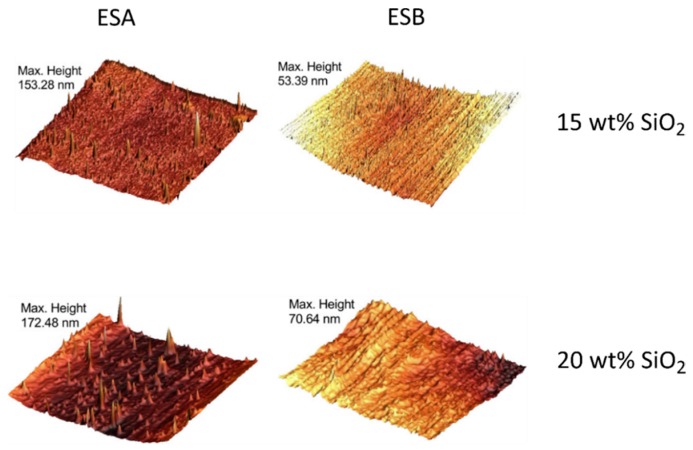
3D atomic force microscopy (AFM) micrographs showing the surface morphology of epoxy nanocomposites (15 and 20 wt % of SiO_2_). The ESA (epoxy silica A) hybrids (**left**) were prepared without 3-glycidyloxypropyltrimethoxysilane (GPTMS), and the ESB (epoxy silica B) hybrids (**right**) were prepared with GPTMS. The peaks represent silica nanoparticles and/or agglomerates, and the SiO_2_ is distributed more homogeneously and less agglomerated when GPTMS is used. Reproduced with permission from Afzal and Siddiqi, *Polymer*; published by Elsevier Ltd., Amsterdam, The Netherlands, 2011.

Afzal and Siddiqui [51] used a similar procedure to that used by Nazir et al. [52] in the preparation of their epoxy–SiO_2_ nanocomposites, the differences being the use of GPTMS as the SCA, a lower pH (2), and a higher temperature for the hydrolysis and condensation reactions (60 °C instead of room temperature). Atomic force microscopy (AFM) was used to investigate the microstructure and surface morphology of the nanocomposites (Figure 7). Inclusion of SiO_2_ in epoxy led to increased roughness of the surface. The peaks in Figure 7 represent the SiO_2_ nanoparticles and show a homogeneous distribution. At higher SiO_2_ loads (above 15 wt %), the SiO_2_ begins to agglomerate. The addition of GPTMS resulted in reduced agglomeration and an improved dispersion of the SiO_2_. Macroscopic phase separation was only observed above 25 and 30 wt % of SiO_2_ for samples treated with and without GPTMS, respectively [51], compared to 20 and 25 wt % for samples treated with and without APTES, respectively [52]. This difference may be attributed to the SCA used, but could also be due to the differences in synthesis conditions. The use of lower pH and higher temperature will promote the hydrolysis of TEOS, resulting in a more network-like open structure, and therefore increased phase mixing. The formation of the silica network was investigated by both Nazir et al. [52] and Afzal and Siddiqui [51], using Fourier transform infrared spectroscopy (FTIR), with the Si–O–Si asymmetric stretching showing an absorption band at 1085 cm^−1^.

**Figure 8:**
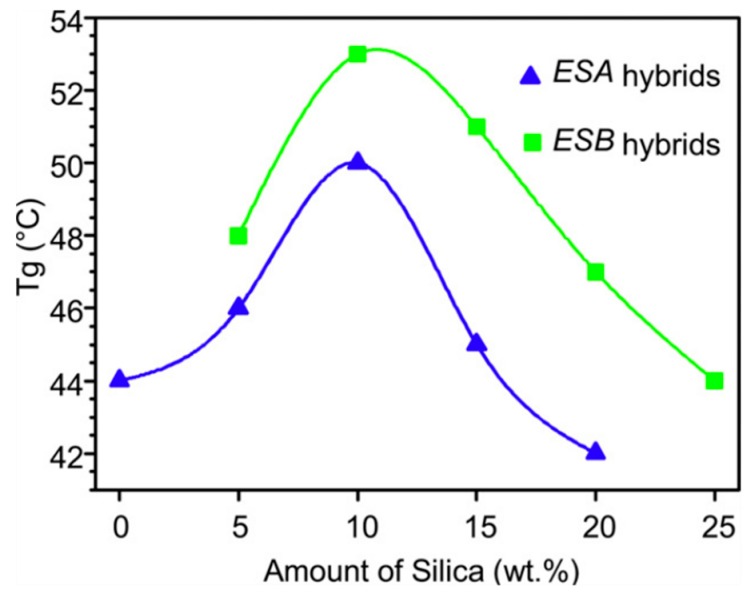
Changes in the glass transition temperature (*T*_g_) with silica content in epoxy nanocomposites. ESA hybrids were not prepared using GPTMS, and ESB hybrids were prepared with GPTMS. Reproduced with permission from Afzal and Siddiqi, *Polymer*; published by Elsevier Ltd., 2011.

Differential scanning calorimetry (DSC) data corroborated the findings from DMTA by Nazir et al. [52], showing an increased *T*_g_ for the epoxy–SiO_2_ nanocomposites (by 6 °C up to 10 wt %) compared to pure epoxy [51]. Afzal and Siddiqui attributed the increase in *T*_g_ to the loss of mobility of the polymer chains around the SiO_2_ nanoparticles, caused by the increased interactions at the interfaces [51]. As with the case of adding APTES, the inclusion of GPTMS also led to a further increase in *T*_g_ for the nanocomposites (by 9 °C up to 10 wt %). Excess amount of SiO_2_ (above 10 wt %), however, showed a decrease in *T*_g_ again (Figure 8), which was suggested to be due to the agglomeration of the nanoparticles, resulting in fewer interactions and fewer immobilized chains [51].

Several other works [36,48,49,50,61] have carried out similar in situ syntheses of epoxy–SiO_2_ and epoxy–TiO_2_ nanocomposites, using various coupling agents (APTES, IPTES, and triethoxysilane-capped trimercaptothioethylamine (TCTMEA)) and reported similar observations for the changes in properties described above. Guan et al. [61] investigated the optical properties of epoxy–TiO_2_ nanocomposite films, and reported over 90% transparency for up to 20 wt % of TiO_2_. The refractive index at 632.8 nm also increased from 1.61 (for pure epoxy) to 1.797 (for 65 wt % TiO_2_). The tensile strength, impact strength, tensile and flexural moduli, and ductility are improved significantly in in situ prepared epoxy–SiO_2_ nanocomposites, compared to pure epoxy [36,48,50]. This toughening of the nanocomposites is attributed to the strong covalent bonds formed at the interfaces between the organic and inorganic networks via the coupling agents, which can withstand external stresses and transfer them to the rigid nanoparticles. However, agglomeration of the nanoparticles in the epoxy can compromise the mechanical properties.

**Figure 9:**
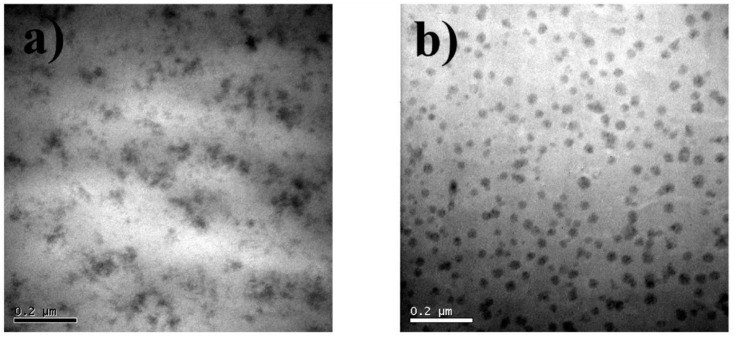
TEM images of the (**a**) epoxy–SiO_2_; and (**b**) epoxy–TiO_2_ nanocomposites prepared with GPTMS as coupling agent. Reproduced with permission from Wu and Hsu, *The Journal of Physical Chemistry C*; published by American Chemical Society, 2010.

Wu and Hsu [59] prepared epoxy–SiO_2_ and epoxy–TiO_2_ nanocomposites (both with 10 wt % of inorganic oxide) using TEOS and tetraethylorthosilicate (TEOT), respectively, as the precursors for the inorganic oxides. GPTMS was used as the coupling agent. For the epoxy–SiO_2_ nanocomposites, they followed a one-step procedure by mixing all the reactants and adding an acid catalyst (HCl) dropwise while stirring. A similar method was used for the epoxy–TiO_2_ nanocomposites, but a mixture of tetraethylorthosilicate (TEOT) and acetylacetone was added dropwise instead of the acid. This was due to the higher reactivity of the TEOT, with the acetylacetone stabilizing the TEOT. Phase separation was observed in samples where GPTMS was not used, whereas the samples prepared with GPTMS were transparent and homogeneous in appearance. TEM images (Figure 9) displayed well-dispersed, non-agglomerated SiO_2_ and TiO_2_ nanoparticles (22 nm average size) in the epoxy resin. The images, however, show distinct TiO_2_ nanoparticles, whereas the SiO_2_ nanoparticles are less distinct in contrast and resemble the IPNs reported by Nazir et al. [52] and Bauer et al. [54]. This could be attributed to the controlled hydrolysis of the TEOT, meaning that the condensation rate is higher, leading to the formation of colloidal TiO_2_ nanoparticles. Meanwhile, TEOS hydrolysis is catalyzed by the acid, leading to the formation of more polymer-like SiO_2_ networks, resulting in the less distinct phase in the TEM image. DSC results confirmed previous observations, with an increase in *T*_g_ observed for both types of nanocomposite [59]. TGA results are consistent with other studies for the epoxy–SiO_2_ nanocomposites, with an increase in thermal stability compared to the pure epoxy. However, the thermal stability of epoxy–TiO_2_ nanocomposites is lower than that of pure epoxy, and is attributed to metal-catalyzed oxidative decomposition pathways [59]. This result is contrary to those reported by Guan et al. [61], where thermal stability increased with increasing TiO_2_ content. Char formation was observed between 400 and 500 °C in both studies.

#### 3.1.3. Application of Ionic Liquids in the Synthesis Procedure

Recently, Donato et al. [62,63,64] used carboxylic and ether ionic liquids (ILs) in both hydrolytic and non-hydrolytic sol–gel processes in the synthesis of epoxy–SiO_2_ nanocomposites. Methylimidazolium-based ILs (organic salts with ionic–covalent crystal structures, e.g., CH_2_CO_2_HMImCl, C_3_H_6_CO_2_HMImCl, C_7_O_3_MImMeS, etc.) can be applied as replacements for conventional volatile solvents, as catalysts for the sol–gel process, or as silica morphology controllers [62]. Due to their selective interaction features and the ability to self-organize, they can act as molecular templates in the sol–gel synthesis of SiO_2_ nanoparticles [63,81], with different ILs resulting in different matrix–filler interface characteristics [62].

Referenced epoxy–SiO_2_ nanocomposites prepared without ILs displayed silica aggregates between 100 and 200 nm in size, while those prepared with C_7_O_3_MImMeS resulted in smaller compact SiO_2_ nanodomains (20–50 nm), forming large loose aggregates with *D*_m_ = 1.7. However, the use of C_1_MImBF_4_ instead resulted in large agglomerates (>200 nm) of dense particles, with *D*_m_ = 3 and poor dispersion [63]. As a result, the nanocomposites prepared with the MeS–anion IL showed strong interfacial interactions, with an increase in the shear storage modulus and decrease in the loss factor, while those prepared with the BF_4_–anion IL showed the opposite (no mechanical reinforcement). However, the nanocomposites prepared with TEOS pre-hydrolyzed using C_10_MImBF_4_ and HCl resulted in the most homogeneous morphology, with 10 nm SiO_2_ domains, leading to the highest shear storage modulus. This is attributed to the IL cation providing physical crosslinking, as the interfacial interaction is weak due to the immiscibility of the IL and the poly(oxypropylene) chains of the epoxy network [63].

**Figure 10:**
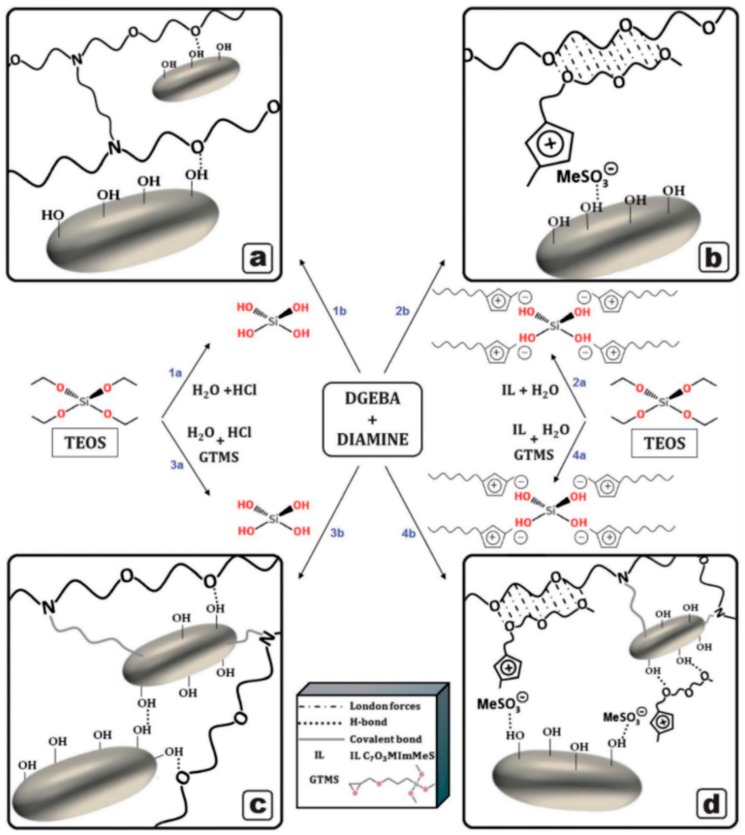
Schematic for various applied synthesis approaches for the preparation of epoxy–SiO_2_ nanocomposites (**a**) without ionic liquid (IL) C_7_O_3_MImMeS; (**b**) with IL C_7_O_3_MImMeS; (**c**) with GPTMS; and (**d**) with GPTMS and IL C_7_O_3_MImMeS. GPTMS is labelled as GTMS in the original figure. The gray platelets represent SiO_2_ nanoparticles. Reproduced with permission from Donato et al., *Journal of Materials Chemistry*; published by The Royal Society of Chemistry, 2012.

SCAs can also be combined with ILs in the in situ synthesis of epoxy–SiO_2_ nanocomposites to tune their mechanical properties. Figure 10 shows a schematic of the various synthesis approaches using ILs and SCAs. The addition of GPTMS in the synthesis led to increased fracture strain and toughness for nanocomposites prepared with both MeS– and BF_4_–anion ILs [64]. On the other hand, the tensile strength decreased initially for small amounts of GPTMS, before increasing with the GPTMS content until 20–30% GPTMS, after which it decreased again [64]. However, combination of the IL and GPTMS in the synthesis decreased the tensile moduli of the nanocomposites, which was attributed to a decrease in the crosslinking density in the organic network. Contrary to the findings reported previously in this review, Donato et al. [64] reported a decrease in *T*_g_ when GPTMS is used along with ILs.

A non-hydrolytic sol–gel approach was also applied to prepare epoxy–SiO_2_ nanocomposites using ILs, with boron trifluoride monoethylamine (BF_3_MEA) complex as the solvent [62]. The reaction in this approach is slower, allowing structure control and avoiding phase separation without the application of a co-solvent [62]. With C_3_H_6_CO_2_HMImCl, the epoxy–SiO_2_ system displayed small, loosely packed agglomerates (10–100 nm), while without the IL the system showed larger agglomerates (500 nm). Similar to the hydrolytic approach, the use of ILs in the non-hydrolytic sol–gel process also resulted in an increase in the shear storage modulus, fracture strain, toughness, and tensile strength. The non-hydrolytic sol–gel approach with ILs also appears to be better suited for glassy epoxy nanocomposites (formed by using more basic amine curing agents, with lower amine equivalent weight) than for rubbery nanocomposites (formed by using less basic amine curing agents, with higher amine equivalent weight) [62]. This is explained to be due to the catalytic effect of the IL (which is slowed down in the non-hydrolytic approach) and the resulting sensitivity to the basicity of the system when the curing agent is added, but further investigation into the effects of the reaction condition is required for these systems.

### 3.2. Polysiloxane Nanocomposites

Polysiloxanes (silicones) are quite prevalent in applications today, for example, in the textile, food, biomedical, aerospace, and electronics industries [82,83,84]. Polydimethylsiloxane (PDMS) is a homopolymer with the general formula H_3_C[Si(CH_3_)_2_O]*_n_*Si(CH_3_)_3_, where *n* is the number of repeating units. The PDMS chains can also contain silanol end groups (Si–OH), forming hydroxy-terminated PDMS (PDMS–OH). The use of transition metal oxide fillers for PDMS has recently attracted interest due to the improvements in the optical and mechanical properties of the nanocomposites, opening new possibilities for applications in optical devices [14]. The unique flexible and rubbery properties of PDMS, along with its thermal stability, have also made it suitable for application as thermally stable rubbers and hydrophobic coatings [66,68,72].

Unlike that of epoxy nanocomposites, the synthesis of most PDMS nanocomposites is done without the use of any coupling agents. This is because hydroxy-terminated PDMS precursors (PDMS–OH) already contain silanol end groups, which allows PDMS to participate in the condensation reaction and be integrated into the inorganic network [68]. Coupling agents are therefore not required to improve compatibility between the organic and inorganic components as they are for epoxy nanocomposites, where there are no silanol end groups in the polymer chains. In addition, the selection of solvents and chelating agents is more important in the synthesis of PDMS nanocomposites containing an inorganic network of a transition metal oxide. This is due to the greater reactivity of the transition metal–alkoxide precursors commonly used. This results in the precipitation of colloidal MO_2_ particles, where M is the transition metal. Therefore, most sol–gel approaches to these nanocomposites are generally two-step procedures—the metal alkoxide is usually stabilized first by the chelating agent or solvent, and then mixed with the PDMS and the hydrolysis and condensation reactions are initiated.

#### 3.2.1. In Situ Synthesis Procedures Using a Chelating Agent for the Transition Metal Alkoxides

One of the earliest applications of the sol–gel approach in the synthesis of PDMS nanocomposites with a transition metal oxide inorganic network was by Glaser and Wilkes, using a chemically controlled condensation method for PDMS modified by TEOS [65]. TEOS and PDMS were initially mixed with isopropanol or THF as a solvent, and then with glacial acetic acid. The mixture was left overnight in N_2_ atmosphere, before TIP was added as the precursor for TiO_2_. This pretreatment with a solvent and glacial acetic acid was to prevent the fast hydrolysis of the TIP. The solution was then mold casted and cured to prepare nanocomposite films. The formation of a Ti–O–Ti network in the PDMS–TEOS hybrid resulted in some improvements in the mechanical properties, notably the increased storage modulus after the glass transition, and the stress at break [65].

**Figure 11:**
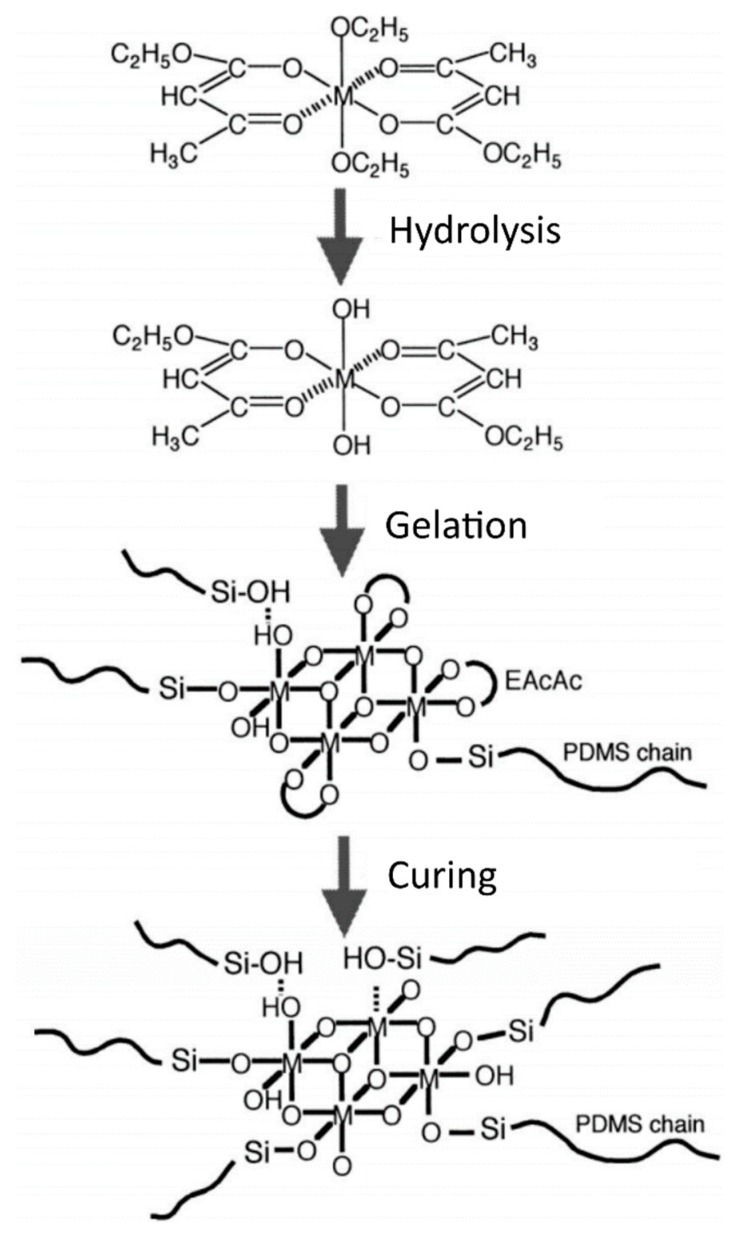
Schematic illustration of the formation of PDMS-based hybrids with metal alkoxide precursors complexed with chelating agent ethyl acetoacetate (EAcAc). Reproduced with permission from Yamada et al. *Journal of Sol–Gel Science and Technology*; published by Kluwer Academic Publishers, 2000.

**Figure 12:**
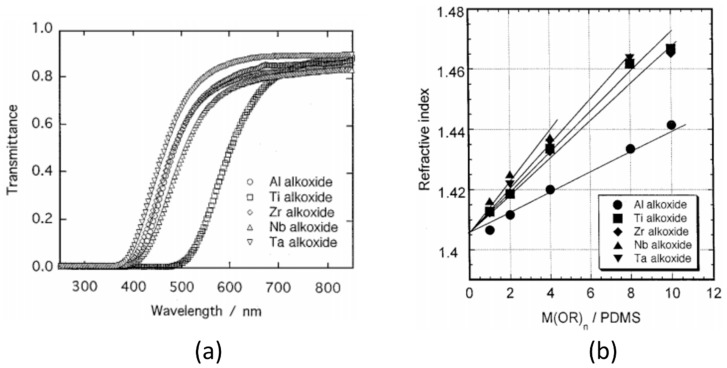
Optical properties of PDMS–O–M hybrids (M = Al, Ti, Zr, Nb or Ta) prepared using M-alkoxides. (**a**) UV-VIS transmittance spectra for hybrids prepared with metal alkoxide to PDMS ratio of 4; (**b**) Change in refractive index with metal alkoxide to PDMS molar ratio for various hybrids. Reproduced with permission from Yamada et al., *Journal of Sol–Gel Science and Technology*; published by Kluwer Academic Publishers, 2000.

Yamada et al. [69] investigated the formation behavior of PDMS hybrids prepared using metal alkoxides of Al, Ti, Zr, Nb, and Ta as the inorganic precursors. The ratio of metal alkoxide to PDMS was varied, and the alkoxides were chemically modified using ethyl acetoacetate (EAcAc) to control the hydrolysis of the reactive alkoxides. The EAcAc was first mixed with the metal alkoxides, followed by addition of ethanol, water, and PDMS. After mixing, the solutions were mold casted and cured at 70 °C for 2 days, followed by post curing at 150 °C for 3 days. ^13^C-NMR and FTIR showed that the EAcAc is bonded to the Al, Ti, and Zr metal alkoxides by substitution of two alkoxy groups, forming a bidentate ligand [69]. For the Ta and Nb alkoxides, however, fewer than 2 alkoxy groups on average were replaced by the EAcAc. Upon addition of water, hydrolysis is initiated and the chelate complex is released. The Al alkoxide, however, formed the strongest chelated complex with EAcAc, and was therefore less subject to the hydrolysis. Chelated complexes are suspected to remain in the hydrolyzed solutions and gels, thus preventing the formation of large inorganic particles. Figure 11 shows an illustration of the formation behavior of these PDMS hybrids when using EAcAc as a chelating agent. The optical properties of these hybrids were also investigated (Figure 12). The refractive indices of the hybrids increased with increasing ratio of metal alkoxide to PDMS (i.e., increasing inorganic content), in the order Al < Zr ≤ Ti < Ta ≤ Nb for the different metal alkoxides [69]. This sequence is attributed to the fact that, with higher valency cations, the chance to form M–O–Si bonds is larger, leading to a more densely crosslinked network between PDMS and the metal oxide. The hybrids were transparent in the wavelength region of visible light (400–700 nm), with an absorption edge at 500 nm, thus appearing yellow in color (except for the Ti–O–PDMS hybrid at 650 nm, appearing red instead). The transmittance was reduced to zero in the UV region for all the nanocomposites (below 400 nm). FTIR showed the emergence of a new absorption band around 930 cm^−1^, which was assigned to the M–O–Si bonds formed by the reaction of the hydrolyzed alkoxides with the Si–OH groups of the PDMS [69].

Shindou et al. [66] prepared PDMS–O–Ti hybrids, using EAcAc as the chelating agent for TIP and ethanol as a solvent. The hybrid films were cured at either 150 °C for 2 h or 300 °C for 6 h. Tapping-mode AFM phase images showed differences in the morphology of the prepared hybrids, depending on the molar ratio of PDMS to TIP and the curing temperature. For the samples cured at 150 °C, low PDMS content (PDMS/TIP < 0.25) showed a continuous phase structure, whereas high PDMS content (PDMS/TIP > 0.35) showed distinct spherical domains of inorganic material (approximately 500–700 nm) in an island-like structure. For the samples cured at 300 °C, however, the images showed homogeneous and featureless surfaces for all PDMS/TIP ratios, indicating homogeneous mixing of the organic and inorganic components in the hybrid [66]. An FTIR absorption band at 960 cm^−1^ disappeared when the sample was heated to 300 °C. This was attributed to a possible coordinate bond between Si and Ti (rather than Si–O–Ti bonds in the hybrid), which is cleaved at higher temperatures (thus resulting in the disappearance of the band) [66]. The hydrophobicity of the hybrids decreased with decreasing molar ratio of PDMS/TIP, as the contact angle of the films with water was reported to decrease with increasing inorganic content in the hybrids.

**Figure 13:**
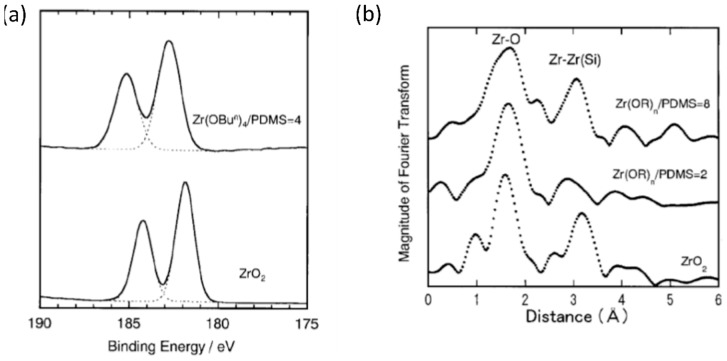
(**a**) Zr*^3d^* XPS spectra of a PDMS–ZrO_2_ hybrid (ZBO/PDMS molar ratio of 4) and ZrO_2_; (**b**) Fourier transforms of extended X-ray absorption fine structure spectroscopy (EXAFS_ spectra for PDMS-ZrO_2_ hybrids (ZBO/PDMS ratio of 8 and 2) and ZrO_2_. Reproduced with permission from Katayama et al. *Journal of the American Ceramic Society*; published by Wiley–Blackwell, 2002.

Katayama et al. [72] prepared PDMS hybrids using zirconium *n*-butoxide (ZBO) and tantalum ethoxide (TE) as precursors for the inorganic components. EAcAc was again used as a complexing agent, and 2-ethoxyethanol was the solvent. A procedure similar to that used by Yamada et al. [69] was applied in the preparation of the hybrid films. X-ray photon spectroscopy (XPS) revealed information on the chemical bonding state of the inorganic components in the hybrids. Figure 13a shows a comparison of the Zr*^3d^* XPS doublet peaks of a PDMS–O–Zr hybrid and ZrO_2_. The similarity of the peaks indicates that Zr was most likely present as an oxide in the hybrid, but the higher shift of the binding energy of the doublet peak also shows that the Zr species were bound to a more electron-attractive species (i.e., the siloxane) [72]. This was corroborated by Fourier transforms of the extended X-ray absorption fine structure spectroscopy (EXAFS) (shown in Figure 13b) as the Zr–Zr(Si) peaks in the hybrids were shifted to shorter distances compared to the corresponding peak for ZrO_2_. This means that the second neighbors of Zr contained other atoms than Zr [72]. The ZrO_2_ nanodomains are then chemically crosslinked to PDMS via Zr–O–Si bonds. High-resolution TEM (HRTEM) images of the hybrids showed inorganic domains of 2–3 nm distributed homogeneously, with the size of the domains increasing with the molar ratio of ZBO/PDMS. Data from SAXS experiments are consistent with the observations from HRTEM. A Guinier analysis indicated a gyration radius of 2.29 nm, which corresponds with the size of the inorganic domains being 2.96 nm, assuming the particles are spherical. FTIR also confirmed the formation of Zr–O–Si bonds between the inorganic domains and the PDMS, with the ZrO_2_ nanodomains behaving as crosslinkers for PDMS chains [72]. DMA revealed an increase in the tensile strength and Young’s modulus of the hybrids, with a larger increase for the PDMS–O–Zr hybrids than for the PDMS–O–Ta hybrids. The tensile strength and Young’s modulus also increased with increasing metal alkoxide to PDMS molar ratio. However, these increases were most prominent at higher temperatures (above 150 °C), which is suspected to be due to increased reaction progress between the PDMS and the hydrolyzed alkoxides. The mechanical properties improved compared to those of PDMS–TEOS hybrid materials. This is suspected to be due to the greater efficiency of the inorganic oxides acting as crosslinkers for PDMS.

**Figure 14:**
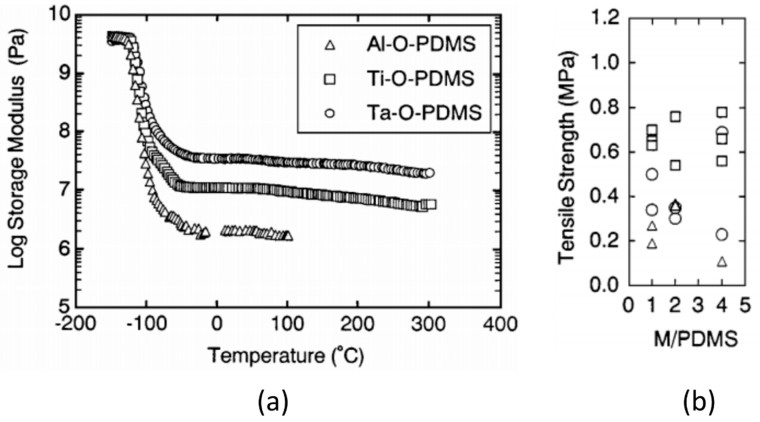
Changes in the (**a**) storage modulus and (**b**) tensile strength, for different PDMS hybrids, as a function of temperature and metal alkoxide to PDMS ratio, respectively. Reproduced with permission from Yamada et al. *Journal of Sol–Gel Science and Technology*; published by Kluwer Academic Publishers, 1998.

Yamada et al. [70,71] also investigated the mechanical properties of PDMS hybrids (prepared using the same procedure described from [69]) earlier, and reported a higher *T*_g_ and storage modulus for the hybrids compared to pure PDMS [70]. The inorganic component was also seen to affect the improvements in the mechanical properties, as shown in Figure 14. The storage modulus at room temperature increased in the order of Al < Ti < Ta as the inorganic network became denser. The tensile strength, however, showed slightly different behavior, and increased in the order Al < Ta < Ti. This was attributed to the degree of interaction between the inorganic component and the PDMS, which is dependent on both the strength of the bond as well as the number of bonds forming [71].

#### 3.2.2. In Situ Synthesis Procedures without Chelating Agents

Almeida et al. [73] prepared PDMS–SiO_2_–MO_2_ hybrids (where M = Ti or Zr) in a slightly different two-step procedure, both with and without a chelating agent. For the samples prepared without a chelating agent, PDMS–OH and the Ti or Zr alkoxides were separately mixed with isopropanol. They were then mixed together simultaneously with TEOS, and the pH was adjusted to 10 or 13. For the samples prepared with a chelating agent, the same procedure was used, but the Ti or Zr alkoxides were mixed with EAcAc instead. After the subsequent curing and heat treatment at 150 °C for 24 h, all the samples were homogeneous and transparent, even those without EAcAc. The lack of precipitation from the transition metal alkoxides in the absence of EAcAc in this case may be attributed to the higher pH, which favors condensation over hydrolysis in the sol–gel reactions, thereby preventing the fast precipitation of TiO_2_ or ZrO_2_ from the hydrolysis of the alkoxides.

Alternatively, the use of isopropanol in the synthesis may also have been responsible for the reduced hydrolysis rate of the alkoxides. This strategy is used in several other works where PDMS hybrids were prepared without chelating agents. The strategy usually involves the use of an alcohol as a solvent—if the same alcohol is a product of hydrolysis of the metal alkoxide, then the reaction equilibrium is shifted to stabilize the alkoxide [14]. Julian et al. [74] prepared PDMS–O–M nanocomposites using dimethyldiethoxysilane (DMDES) as the PDMS precursor, and metal alkoxides of Al, Ge, Sn, Ti, Zr, Nb, and Ta. Isopropanol or propanol was used as the solvent for the metal alkoxides. The arrangement and length of the siloxane chains were affected by the type of metal alkoxide used in the hybrids, based on results from FTIR and ^29^Si MAS (magic angle spinning) NMR. In addition, the bands for symmetric Si–O–Si stretching vibration in the Raman spectra were also shifted to lower wavenumber in the hybrids, indicating a more rigid environment for the PDMS chains. The PDMS hybrid system containing Ge most likely consisted of short PDMS chains with a few siloxane units (4–5), while the systems containing Ti, Zr, and Al consisted mostly of long PDMS chains, and those containing Ta, Nb, and Sn had an intermediate structure. The crosslinking effect of the transition metals (via M–O–Si bonds, as evidenced by FTIR) appears to be strongest for Ta- and Nb-containing hybrids, as shown by the largest increases in *T*_g_ [74].

**Figure 15:**
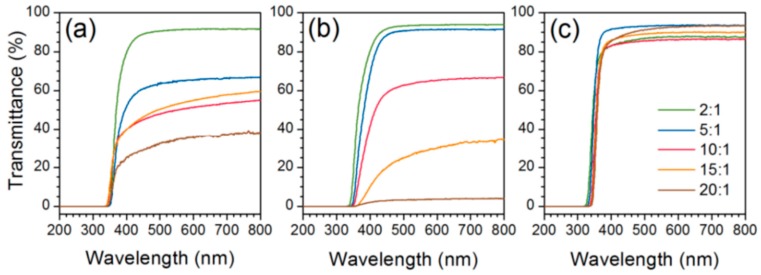
UV-VIS spectra of PDMS–TiO_2_ hybrid films for different TIP/PDMS–OH molar ratios (shown in the legend), using PDMS–OH precursors with viscosities of (**a**) 25; (**b**) 65; and (**c**) 750 cSt, corresponding to average molar masses of 2100, 4000, and 20,000 g mol^−1^, respectively (calculated based on experimental measurements from [85]). Reproduced with permission from Dalod et al. *Nanomaterials*; published by MDPI, 2017.

**Figure 16:**
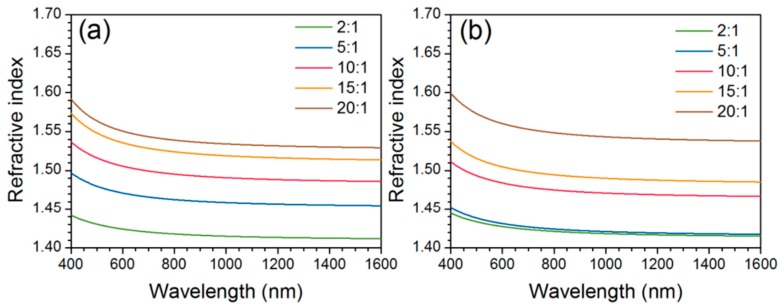
Changes in the refractive indices for PDMS–TiO_2_ hybrid films for different TIP/PDMS–OH molar ratios (shown in the legend), using PDMS–OH with viscosities of (**a**) 25 cSt; and (**b**) 65 cSt, corresponding to average molar masses of 2100 and 4000 g mol^−1^, respectively (calculated based on experimental measurements from [85]). Reproduced with permission from Dalod et al. *Nanomaterials*; published by MDPI, 2017.

More recently, Lu and Mullins [67] used a non-aqueous sol–gel procedure for PDMS–TiO_2_ nanocomposites. The hydrolysis of TIP was reduced by mixing with isopropanol before being mixed with PDMS. This procedure was then adapted by Dalod et al. [14] with anhydrous isopropanol instead, and with different viscosities of a PDMS–OH precursor. No chelating agent was used in either of these works. Presence of FTIR bands attributed to Ti–O–Si units and Ti–O–Ti units, as well as the absence of the broad band for –OH groups, confirmed the full reaction of the –OH groups of the PDMS–OH precursor, and the formation of TiO_2_ nanodomains crosslinked with PDMS. At high TiO_2_ contents, additional bands corresponding to amorphous TiO_2_ were observed from Raman spectroscopy, indicating the formation of larger amorphous TiO_2_ domains or particles. These TiO_2_ domains were calculated to be 3.8 nm on average, based on SAXS measurements. The correlation length between the amorphous inorganic domains decreased with increasing content of TiO_2_, indicating that the particles are more densely packed (assuming constant particle size). The contact angle with water decreased with increasing TiO_2_ content, showing a reduced hydrophobicity in the hybrids (as seen similarly from the results reported by Shindou et al. [66]). The optical properties of the hybrids were also measured, showing good transparency in the visible range of the spectrum for hybrid films with low TiO_2_ content. However, at higher TiO_2_ contents, the transparency dropped significantly for the films containing PDMS with 25 and 65 cSt viscosity, due to scattering of light on the rough surface of the samples (Figure 15). The transmittance was also reduced to zero in the UV region (below 320 nm), most likely due to the absorbance of UV by the TiO_2_ nanoparticles. With increasing amount of inorganic content, the refractive index increased for the PDMS–TiO_2_ hybrid films (Figure 16). The Abbe number decreased with increasing TiO_2_ content, showing increased dispersion in the visible region of the light spectrum [14]. The hybrid films were flexible and stiff below and above approximately 5 vol % TiO_2_, respectively. This observation was corroborated through dynamic mechanical analysis, where the shear storage moduli (G’) of the hybrid films were measured from 2 to 220 MPa at room temperature, increasing the amount of titanium precursor. At high titania incorporation, the increased fractal dimension as well as the low correlation length (2 nm) measured by SAXS may indicate percolation (titania nanodomains are connected to each other throughout the material), which could explain the drastic increase of G’.

#### 3.2.3. PDMS–SiO_2_ Nanocomposites by Swelling Techniques

While transition metal-based PDMS hybrids have attracted more interest in recent years due to the possibility to expand their range of applications, silica has traditionally been used to reinforce the mechanical properties of PDMS, particularly to increase the tensile strength, in most commercial applications [2,76]. The swelling technique used for epoxy–SiO_2_ nanocomposites by Bauer et al. [54] and Matějka et al. [55] is also often used for the synthesis of PDMS–SiO_2_ nanocomposites. Mark and Pan [5] pioneered PDMS–SiO_2_ nanocomposites using a sol–gel procedure as early as 1982, with cured PDMS films (from vinyl-terminated and hydroxy-terminated chains) being swollen in TEOS, followed by addition of glacial acetic acid, water, and a phase-transfer catalyst ((*n*-Bu)_4_PBr). The hybrids were then extracted in THF and dried under vacuum. The formation of SiO_2_ was found to be difficult in the absence of the catalyst. Stress–strain measurements revealed that the in situ prepared nanocomposites showed increased toughness [5].

Yuan and Mark [77] applied a different double-swelling procedure, where the cured PDMS films were swollen first in TEOS, and then in diethylamine (DEA) as a catalyst. This process was repeated with a different concentration of DEA, resulting in bimodal particle distributions in the nanocomposite. The particle size could be controlled by the concentration of the catalyst, as it affects the rates of the hydrolysis and condensation reactions. TEM and SAXS measurements showed that the SiO_2_ particles formed with a lower concentration of DEA were smaller (20–25 nm) than those formed with a higher concentration of DEA (160 nm), which is expected based on the type of catalyst used (DEA is basic, thereby favoring the condensation reactions more). The in situ approach also resulted in improved dispersion quality, compared to a traditional blending with pre-synthesized SiO_2_ nanoparticles. Smaller filler particles imparted greater ultimate tensile strength to the nanocomposites, although larger particles improved extensibility more significantly [77]. In addition, several hybrids were prepared in situ with surface modified SiO_2_ using dimethyldiethoxysilane (DMDEOS) and vinyl-terminated PDMS precursors. This approach allowed the nanocomposite to remain hydrophobic despite the inclusion of hydrophilic SiO_2_ nanofillers. Breiner et al. [78] also showed that the particle size of the SiO_2_ filler was dependent on the molecular weight (*M*_c_) of the PDMS chains, with shorter chains (low *M*_c_) resulting in smaller particles due to possible constraining effects.

Dewimille et al. [76] also synthesized PDMS–SiO_2_ nanocomposites by swelling cured PDMS films in TEOS, along with either dibutyltin diacetate (DBTDA) or dibutyltin dilaurate (DBTDL) as a catalyst. Hybrid films were also prepared using DEA, similar to Yuan and Mark [77]. From the TEM images, differences in the inorganic structures of the hybrids were observed depending on the type of catalyst used. Particles generated using DEA were larger and more spherical than those generated by DBTDL. SAXS measurements indicated that the DEA-catalyzed systems contained uniformly dense objects with smooth surface fractal dimensions (*D*_s_ = 2), with the particle radius being calculated to around 15 nm (consistent with TEM images), while the tin-catalyzed systems contained more polymeric inorganic structures instead. This is as expected, since DEA is a more basic catalyst than both DBTDA and DBTDL.

**Figure 17:**
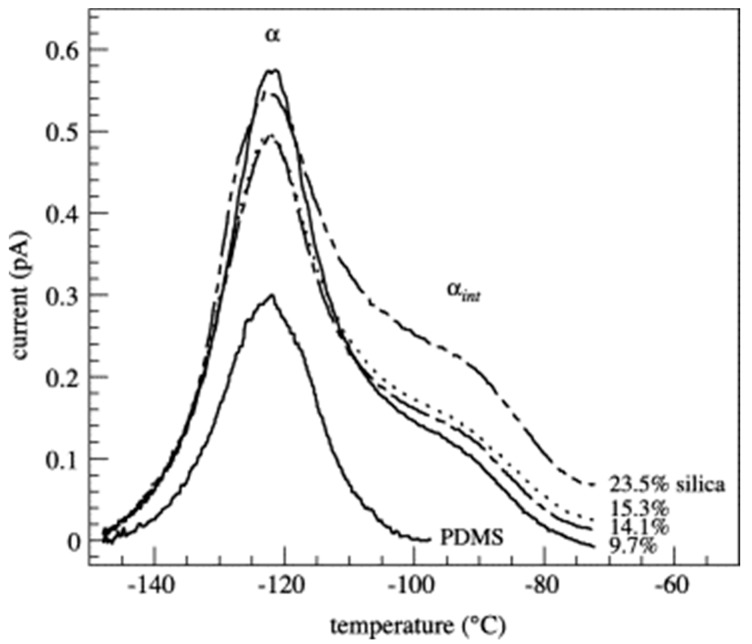
Thermally stimulated depolarization currents (TSDC) thermogram for PDMS and PDMS–SiO_2_ nanocomposites in the glass transition region. Reproduced with permission from Fragiadakis et al. *Polymer*; published by Elsevier Ltd., 2005.

Fragiadakis et al. [75,86] used the same procedure as Dewimille et al. [76] with DBTDA and hydride-terminated PDMS and investigated the glass transition and molecular dynamics of the hybrids. While *T*_g_ is not seen to be affected by the SiO_2_ content in PDMS, the shape of the step up in heat capacity changes, which indicates contribution of the SiO_2_ in the high temperature side. Thermally stimulated depolarization currents (TSDC) were used to characterize the glass transition further, and for the hybrids containing SiO_2_ two different α relaxations were present (Figure 17). The first was the primary relaxation associated with the glass transition of the amorphous bulk PDMS (at a temperature which is in good agreement with the DSC measurements), and the second (α_int_ in Figure 17) was assigned to the α relaxation of the PDMS chains in an interfacial layer close to the SiO_2_ [75,87]. The double structure of the α-relaxation was attributed to a gradual slowing down of the chain mobility at the interface with the SiO_2_ nanoparticles. The range of this interfacial region was calculated to be about 3 nm (based on the dielectric strengths of the two components of the relaxation) [86]. Rajan et al. [79] investigated the optical properties of PDMS–SiO_2_ films prepared using the swelling sol–gel approach with either DBTDL/DBTDA catalysts, or with ammonia. UV-VIS transmittance spectra showed over 90% transparency for PDMS–SiO_2_ nanocomposites prepared with the neutral catalysts. However, for nanocomposites prepared with ammonia, transparency was lower (70% for 4 wt % SiO_2_) and the samples became opaque with higher SiO_2_ content (18.5 wt %). This may be attributed to the larger colloidal particles or aggregates that formed under basic conditions during the sol–gel process.

## 4. Alternative Methods for the In Situ Preparation of Nanocomposites

While this review has so far focused on nanocomposites based on the epoxy and PDMS systems to demonstrate syntheses of SiO_2_ and transition metal oxide nanoparticles using the sol–gel method, the method is versatile and can be applied to several other polymer systems. SiO_2_ and TiO_2_ nanoparticles have been successfully synthesized and incorporated in, among other polymeric systems, poly(ethylene oxide), polypropylene, polyimide, and polyaniline, using alkoxide precursors [88,89,90,91,92,93]. In addition, the sol–gel method can also be combined with other techniques (e.g., electrospinning) to form different structures, such as hybrid nanofibers [94]. The first part of Table 2 shows examples of the various hybrid systems that have been prepared using sol–gel methods. In addition, several other techniques and methods are used for miscellaneous polymer–inorganic nanocomposites. The second part of Table 2 includes selected examples of such cases, where methods that are not based on the sol–gel process are described briefly below to illustrate the chemistry behind the different procedures.

Balci et al. [95] prepared Si nanocrystal organic hybrid films using a single batch solution synthesis. SiCl_4_ was reduced in a solution of potassium cyclopentadiene, and the resulting Si nanocrystals were surface-terminated using either 1-octanol, oleic acid, acrylic acid, or ethylene glycol. The reaction is based on potassium cyclopentadiene acting as a nucleophile towards the silyl halide, forming Si clusters [96]. The following reaction of SiCl_4_ on the surface with OH groups of surface capping agent produces HCl [97,98]. In the presence of HCl with excess -OH groups from surface termination groups, H_2_O is formed [42]. In addition, a possible side reaction of the cyclopentadiene with the surface termination groups can occur, such as the Diels–Alder reaction with acrylic acid, which results in 5-Norbornene-2-carboxylic acid [99]. The exact nature of the organic network is unknown, and it is suspected that the network is formed by the polymerization of cyclopentadiene into dienophilic oligomers, possibly including polysiloles from the Diels–Alder reaction [100]. Different surface termination groups will have different resulting polymeric structures than the cyclopentadiene group, which will also affect the size distribution of Si nanocrystals and optoelectronic properties of the hybrid film. The mean size of the Si nanocrystals (2–10 nm) was dependent on the type of surface termination, with acrylic acid and 1-octanol causing the largest mean sizes, and ethylene glycol causing the smallest mean sizes. The hybrid films exhibited white light photoluminescence at room temperature when excited by UV light, which was stable after storage over six months. Ethylene glycol terminated Si nanocrystals showed the largest red shift in photoluminescence.

Du et al. [106] incorporated CdS nanoparticles in situ in polystyrene (PS), and showed that the density and size of the CdS nanoparticles can be controlled by altering the sulfonate content in PS. A higher sulfonate content resulted in smaller and more uniform nanoparticles. The microstructure of the PS network, which includes clustering of ionic groups such as ${\mathrm{SO}}_{3}^{-}$, was used to confine the nanoparticles formed. The nanoparticles were synthesized by the reaction between S^2−^ ions released from the decomposition of thioacetamide (TAA) reacted with Cd^2+^ ions from the acetate.

Zhang et al. [31] applied an in situ solvothermal synthesis procedure for preparing epoxy–TiO_2_ nanocomposites, using GPTMS as a coupling agent and TBO as the TiO_2_ precursor. TBO, ethanol, HCl, and GPTMS were added to DGEBA (preheated to 80 °C), and the homogeneous mixture was transferred to an autoclave. The sealed vessel was then placed in an oven at 100 °C for 3 h. The autogenous pressure inside the vessel increased beyond atmospheric pressure as the temperature is increased, increasing the solubility and reactivity of the reactants, resulting in the hydrolysis and condensation of the TBO. The nanocomposites showed high transparency (above 80% in the visible region of the spectrum) and improved absorbance of UV light (300–420 nm), which increased with increasing TiO_2_ content. The films also showed increased hydrophobicity (based on the contact angle with water) when GPTMS was used, indicating successful crosslinks between the nanofiller surface and the polymer chains.

Liu et al. [107] prepared epoxy–SiO_2_ nanocomposites using reverse (water in oil) microemulsions, consisting of epoxy resin as the oil phase and an aqueous ammonia solution as the water phase. TEOS was added to the prepared microemulsion and the in situ polymerization of TEOS within the water phase formed the nanoparticles. In this method, appropriate surfactants should be used to stabilize the microemulsion and control the water solubilization in the oil phase (epoxy). Since ammonia was used in the water phase, the condensation reaction of the TEOS will be catalyzed and should form colloidal particles.

## 5. Prospects of In Situ Hybrid Materials

Hybrid materials are an attractive choice for applications in multiple areas due to the unique combination of properties that can be achieved from the organic and inorganic components. Proper selection of the polymeric matrix and the inorganic fillers (oxides, metals, sulphides, etc.), and the synthesis routes and conditions can allow one to tailor these properties as required, and design new materials and compounds. Earlier in this work, it has been shown how the in situ generation of inorganic oxide nanoparticles or nanodomains can result in improvements in the mechanical properties (tensile strength, Young’s modulus, storage modulus, ductility, etc.), the thermal properties (*T*_g_ and thermal stability), as well as the optical properties (transparency and refractive index) for epoxy- and PDMS-based nanocomposites. Several works have shown improvements in other areas as well, such as the dielectric properties, where the nanocomposites were prepared using conventional ex situ methods [18,53,108,109]. Since it is known that ex situ preparation techniques are not optimal for achieving an agglomerate-free and homogeneous dispersion of particles, there is potential room for improving these materials further, using in situ techniques to synthesize the nanocomposites.

Epoxy–SiO_2_ nanocomposites have been extensively investigated for applications in electrical insulation, flame-retardant and anticorrosive coatings, encapsulation, adhesives, laminates, aerospace parts, etc. [18,32,51]. Transition metal oxides (TiO_2_, Nb_2_O_5_, Ta_2_O_5_, ZrO_2_, etc.) in epoxy and PDMS have also shown great potential for optical applications (since they can increase the refractive indices for both polymers while maintaining the transparency), such as in lenses, transparent coatings, or LED encapsulation [14]. TiO_2_ can also introduce photocatalytic properties to polymer materials and improve resistance to UV degradation. A number of studies have also been carried out on PDMS-based hybrids (with TiO_2_, CaO, and SiO_2_ as the inorganic component) for use as bioactive materials in the human body to assist bone reparation and formation via apatite formation [33,110,111].

Other polymer nanocomposites can be used similarly in diverse applications. Table 3 shows selected examples of such potential applications of different polymer–oxide nanocomposites. Several polymers (e.g., Nafion, PVA, PVDF, polyimide, etc.) that are commonly applied as membranes in high-temperature proton exchange membrane (PEM) fuel cells have shown improved properties upon the inclusion of oxide fillers [3,112,113]. Improved cell performances have been observed for these nanocomposite membranes at higher temperatures (110–120 °C) and lower relative humidity (80–90%). The use of in situ sol–gel methods in the preparation of these nanocomposites, rather than ex situ casting methods, has led to better dispersion of the nanoparticles and, consequently, a better performance of the material [105]. PEG- and PEO-based nanocomposites may be applied as solid electrolytes for Li–ion batteries, thanks to increased ionic conductivity and thermal stability [88,114]. Poly(ethylene terephthalate) (PET) with ZnO nanofibers prepared through a combination of sol–gel and electrospinning has shown high luminescence capacity, enabling its use in photocatalysts, gas sensors, light emitters, and solar cells [94]. Biopolymers (e.g., chitosan, alginate, etc.) can also be combined with inorganic oxides to form nanocomposites that are used as biosensors and drug-delivery agents [3,115].

## 6. Conclusions

The synthesis of inorganic oxide nanoparticles in situ directly in the polymer matrix is an alternative approach to the preparation of polymer nanocomposites, contrary to nanocomposites processed using traditional ex situ mixing techniques. The use of the sol–gel method in the in situ approach has been shown to be robust and flexible, offering improved control over the morphology of the inorganic structures formed and, therefore, the ability to tailor the properties for desired applications. Perhaps the greatest advantage provided by the sol–gel method is the ability to form Class II hybrid materials with an improved quality of dispersion of the inorganic components. Consequently, improvements are observed in the optical, thermal, and mechanical properties of the nanocomposites, which will broaden the scope of applications for these materials.

In the present review, an appraisal of the sol–gel method for the synthesis of SiO_2_ and M_x_O_y_ (where M is a transition metal) nanoparticles in epoxy- and PDMS-based nanocomposites, respectively, has been performed. The nanoparticles are formed via the hydrolysis and subsequent condensation of metal alkoxide precursors inside the monomer resins, followed by the curing of the resins to form the nanocomposite. For the epoxy nanocomposites, the use of surface functionalization (e.g., SCAs or ILs), acid catalysts (e.g., TSA, DBTDL), higher synthesis temperatures, and a two-step procedure with pre-hydrolyzed TEOS have all contributed to smaller and more open nano-SiO_2_ structures, with reduced agglomeration and a bicontinuous phase morphology in the nanocomposites. These changes in the structure of the inorganic domains have led to increases in the glass transition temperature, the thermal stability, the mechanical strength, and the storage modulus, and a decrease in the loss factor. For the PDMS nanocomposites, due to the increased reactivity of the transition metal alkoxide precursors, the use of complexing agents (e.g., EAcAc), or an appropriate solvent (e.g., isopropanol) is significant in preventing phase separation or the formation of colloidal MO_2_. The formation of MO_2_-like nanodomains that are crosslinked with PDMS chains resulted in increased light transmittance, UV absorbance, refractive index, tensile strength, and shear storage modulus in the nanocomposites.

While several alternatives exist for synthesizing inorganic oxide nanoparticles in situ in the polymer, none are more ubiquitous than the sol–gel method, which is applied not only for the two polymer systems focused on in this review, but also for various other polymer–inorganic oxide nanocomposites. The improvements in the properties observed for these hybrid materials make them attractive choices for application in coatings, packaging materials, nanodielectrics, optical devices, and photovoltaics. The next step in the development of these nanocomposites will be to adapt the in situ methods to enable fabrication of the materials at a larger and economic scale, making the leap from the laboratory to the industry.

## Figures and Tables

**Table 1:** Selected examples of the precursors, surface modification and solvents used in the in situ synthesis of metal oxide nanoparticles via sol–gel processes in epoxy and polydimethylsiloxane (PDMS) nanocomposites.

Polymer System	Inorganic Component	Inorganic Precursor	Surface Modification	Solvent	Reference
Epoxy ^1^	SiO_2_	TEOS	-	-	[54]
			-	Isopropanol	[55,56,57]
			APTES	Ethanol	[38]
				-	[52]
			GPTMS	-	[51]
			IPTES	-	[48]
				Ethanol	[49]
		TEOS, DPTEOS ^2^		-	[58]
		APTES	APTES	DMF	[36]
	SiO_2_, TiO_2_	TEOS, TEOT ^3^	GPTMS	Acetylacetone	[59]
	TiO_2_	TIP		-	[60]
		TBO ^4^	TCTMTEA ^5^	Anhydrous THF	[61]
	SiO_2_	TEOS	-	Ionic liquids ^6^	[62,63,64]
PDMS	SiO_2_, TiO_2_	TEOS, TIP	-	THF and isopropanol	[65]
	TiO_2_	TIP	-	Ethanol	[66]
			-	Isopropanol	[14,67]
	SiO_2_, TiO_2_	TIP, TEOS, MTES ^7^	-		[68]
	M_x_O_y_^8^	M(OR)_n_^8^	-	Ethanol	[69,70,71]
	ZrO_2_, TaO_2_	ZBO, TE ^9^	-	2-ethoxyethanol	[72]
	SiO_2_–TiO_2_/ZrO_2_	TEOS, TIP, ZP ^10^	-	Isopropanol	[73]
	M_x_O_y_^11^	M(OR)_n_^11^	-	Ethanol and isopropanol	[74]
	SiO_2_	TEOS	-	-	[5,75,76,77,78,79,80]
			DMDEOS ^12^	-	[77]

^1^ Molecular weight of the amine curing agent varies (between 230–1970); ^2^ Diethylphosphatoethyltriethoxysilane; ^3^ Tetraethylorthotitanate; ^4^ Titanium (IV) butoxide; ^5^ Triethoxysilane-capped trimercaptothioethylamine (TMTEA). Acts as both coupling and curing agent; ^6^ CH_2_CO_2_HMImCl, C_3_H_6_CO_2_HMImCl, andC_7_O_3_MImMeS; ^7^ Methyltriethoxysilane; ^8^ M = Al, Ti, Ta, Zr, Nb; ^9^ Zirconium (IV) *n*-butoxide (ZBO), Tantalum (IV) ethoxide (TE); ^10^ Zirconium propoxide; ^11^ M = Al, Ge, Sn, Ti, Zr, Nb, Ta; ^12^ Dimethyldiethoxysilane.

**Table 2:** Selected examples of miscellaneous polymer–inorganic nanocomposites prepared using various in situ synthesis procedures. The first part of the table shows nanocomposites prepared using sol–gel techniques, and the second part shows nanocomposites prepared using other in situ approaches.

Polymer	Inorganic Component	Inorganic Precursor	Method	Reference
Poly(ethylene oxide)	SiO_2_	TEOS	Sol–gel	[88]
Polypropylene	TiO_2_	TBO		[89,90]
	SiO_2_	TEOS		[91]
Polyimide	SiO_2_			[92]
Polysulfone	TiO_2_	TBO		[101]
Poly(vinyl-co-acetate)	SiO_2_	TPO ^1^		[102]
Polyethylene-octene	SiO_2_	Si(OH)_4_		[103]
Polyaniline	SiO_2_	TEOS	Combination of sol–gel and in situ polymerization	[93]
Poly(ethylene terephthalate)	ZnO	ZA ^2^	Combination of sol–gel and electrospinning	[94]
	SiO_2_	TEOS	Sol–gel	[104]
Nafion	TiO_2_	TBO		[105]
Unidentified ^3^	Si	SiCl_4_	Solution-based reduction	[95]
Polystyrene	CdS	CA ^4^	In situ precipitation	[106]
Epoxy	TiO_2_	TBO	Solvothermal synthesis	[31]
	SiO_2_	TEOS	Reverse microemulsion	[107]

^1^ Tetrapropoxysilane; ^2^ Zinc acetate dehydrate; ^3^ Polymerization of cyclopentadiene into dienophilic oligomers, and possible polysilole, is suspected; ^4^ Cadmium acetate.

**Table 3:** Selected examples of applications and relevant features for several polymer–inorganic oxide nanocomposites.

Application	Nanocomposite System	Features	Reference
Electrical insulation	Epoxy–SiO_2_ Epoxy–TiO_2_	Increased dielectric breakdown strength Decreased complex permittivity Increased *T*_g_ Increased strength, toughness, ductility	[16,18,108,116,117,118,119,120]
	PE ^1^–MgO PE–Al_2_O_3_	Decreased DC conductivity Increased dielectric breakdown strength Decreased space charge accumulation Decreased complex permittivity	[121,122,123,124,125]
	PI ^2^–SiO_2_	Decreased electrical conductivity Increased scratch hardness Increased strength	[126]
Fuel cells	Nafion–TiO_2_ Nafion–SiO_2_	Increased water uptake Decreased resistivity Increased *T*_g_ Increased degradation temperature	[105,127,128]
	PVA ^3^–SiO_2_	Increased liquid retention Increased proton conductivity Higher ion-exchange capacity	[129,130]
	PVDF ^4^–Al_2_O_3_	High proton conductivity High thermal stability Low methanol permeability Increased water uptake	[131]
Coatings	Epoxy–SiO_2_	Increased flame retardance High *T*_g_ Good thermal stability	[132]
	PDMS–TiO_2_–SiO_2_	Transparent and hydrophobic Increased hardness	[68]
	Epoxy–SiO_2_ Epoxy–Fe_2_O_3_	Improved corrosion resistance Increased Young’s modulus	[133]
	PDMS–PVA–ZnO	Decreased hydrophobicity Reduced contamination by fluorescent biomarkers in biosensors	[134]
	Epoxy–PANI ^5^–ZnO	Antifouling and antibacterial properties	[135]
Bioactive materials	PDMS–CaO–SiO_2_–TiO_2_	Increased Young’s modulus High apatite-forming ability High extensibility High strength	[33,111]
	PDMS–CaO–SiO_2_	High apatite-forming ability Decreased release of silicon in body fluids Mechanical properties analogous to those of human cancellous bones	[110]
Solid electrolytes	PEO ^6^–SiO_2_	Increased Li^+^ transference number Increased *T*_g_	[88]
	PEG–PU/PAN ^7^ with TiO_2_	Good thermal stability Increased ionic conductivity	[114]
Ultrafiltration	PS^8^–TiO_2_	Increased hydrophilicity Increased permeability Increased *T*_g_	[101]
Electromagnetic interference shielding (EMI)	PANI–SiO_2_	Increased EMI shielding effectiveness Increased thermal stability	[93]

^1^ Polyethylene; ^2^ Polyimide; ^3^ Poly(vinyl acetate); ^4^ Poly(vinylidene fluoride); ^5^ Polyaniline; ^6^ Poly(ethylene oxide); ^7^ Poly(ethylene glycol) –polyurethane/poly(acrylonitrile); ^8^ Polystyrene.
